# Impulsivity Traits in Parkinson's Disease: A Systematic Review and Meta‐Analysis

**DOI:** 10.1002/mdc3.13839

**Published:** 2023-07-26

**Authors:** Fabio Giovannelli, Gioele Gavazzi, Chiara Noferini, Pasquale Palumbo, Maria Pia Viggiano, Massimo Cincotta

**Affiliations:** ^1^ Department of Neuroscience, Psychology, Drug Research and Child's Health (NEUROFARBA), Section of Psychology University of Florence Florence Italy; ^2^ European Laboratory for Non‐Linear Spectroscopy (LENS) Sesto Fiorentino Italy; ^3^ Unit of Neurology of Prato, Cerebrovascular and Neurodegenerative Disease Area of the Department of Medical Specialties Central Tuscany Local Health Authority Prato Italy; ^4^ Unit of Neurology of Florence, Cerebrovascular and Neurodegenerative Disease Area of the Department of Medical Specialties Central Tuscany Local Health Authority Florence Italy

**Keywords:** Parkinson's disease, impulse control disorders, impulsivity, Barratt Impulsiveness Scale

## Abstract

**Background:**

In Parkinson's disease (PD), impulsivity as a personality trait may be linked to the risk of developing impulse control disorders (ICDs) during dopaminergic therapy. However, studies evaluating differences in trait impulsivity between patients with PD and healthy controls or between patients with PD with and without ICDs reported partly inconsistent findings.

**Objectives:**

We conducted a systematic review and meta‐analysis (Preferred Reporting Items for Systematic Reviews and Meta‐Analyses) of studies comparing Barratt Impulsiveness Scale (BIS‐11) scores between patients with PD and healthy controls and between patients with PD with and without ICDs.

**Methods:**

Eligible studies were identified through a systematic search in 3 databases. Mean differences with 95% confidence intervals (CIs) for BIS‐11 total and subscale scores were separately calculated for studies comparing patients with PD and healthy controls and patients with PD with and without ICDs. Meta‐regressions were performed to explore sources of heterogeneity (percentage of men, age, disease duration, and levodopa equivalent daily dose).

**Results:**

A total of 40 studies were included in the quantitative analyses. BIS‐11 total scores were significantly higher in patients with PD compared with healthy controls (mean difference 2.43; 95% CI, 1.03, 3.83), and in patients with PD with active ICDs compared with patients without ICDs (6.62; 95% CI, 5.01, 8.23). No significant moderators emerged by meta‐regression analyses.

**Conclusions:**

The present meta‐analysis supports that impulsivity, as a personality trait, may characterize patients with PD, even in the absence of ICDs. Moreover, these data corroborate findings of clinical studies reporting higher levels of trait impulsivity in PD patients with ICDs compared with patients without ICDs.

Impulsivity is a multidimensional concept relevant either in the description of normal individual differences in personality or as a maladaptive factor characterizing a variety of pathological conditions with a lack of behavioral control as a common feature.^1^, ^2^, ^3^ An impulsive personality trait may be defined as a tendency toward rapid and unplanned reactions to internal or external stimuli without regard to the negative consequences.^4^ This definition incorporates the following 3 core elements of impulsivity: (1) decreased sensitivity to immediate negative feedbacks; (2) rapid, unplanned reactions to stimuli before complete processing of information; and (3) lack of regard for long‐term consequences of behavior.^4^ These aspects of impulsivity traits are configured as peculiar features of impulse control disorders (ICDs).^3^, ^4^

ICDs, which include pathological gambling, hypersexuality, and compulsive eating and shopping and related behaviors such as punding, hoarding, hobbyism, and compulsive medication overuse are commonly reported in patients with Parkinson's disease (PD) during dopaminergic therapy.^5^, ^6^ ICDs are associated with greater functional impairment, decreased quality of life, and increased caregiver burden and represent a critical issue for the clinical management of patients with PD. ICDs may result from the interaction between predisposing factors (ie, demographic, psychological, clinical, and genetics factors) and dopaminergic medication.^7^, ^8^

Therefore, it has been suggested that PD patients with high levels of impulsivity, along with other personality characteristics such as novelty seeking, can be at higher risk for developing ICDs.^9^, ^10^, ^11^ In keeping with this hypothesis, higher impulsivity, as assessed by self‐report questionnaire, was reported in PD patients with ICDs compared with patients without ICDs.^9^, ^12^, ^13^, ^14^ Moreover, evidence of higher levels of trait impulsivity have been reported in patients with PD when compared with healthy controls even in the absence of ICDs.^14^, ^15^ However, some studies did not report differences either from the comparison between patients with PD and healthy controls^16^, ^17^ or between patients with and without ICDs.^18^, ^19^ Therefore, whether impulsivity may represent a main vulnerability factor for the development of ICDs during dopaminergic treatment still remains an open question.

The overall goal of the present study was to investigate impulsivity as a personality trait in patients with PD using a meta‐analytic approach to the current literature. To this end, we performed 2 separate meta‐analyses aimed to verify: (1) whether impulsivity traits are higher in patients with PD compared with age‐matched healthy individuals and (2) whether and to what extent impulsivity traits differ between PD patients with and without ICDs. Moreover, we explored the possible influence of demographic and clinical factors through meta‐regression analyses. We used the total score of the Barratt Impulsiveness Scale (BIS‐11) as a primary measure. BIS‐11 is the most administered self‐report questionnaire to assess impulsive personality traits.^20^ Recently, a relationship between BIS‐11 score and awareness of motor intention has been found both in healthy subjects^21^, ^22^ and patients with PD.^23^

## Methods

### Search Strategy and Selection Criteria

We performed a systematic and comprehensive literature search up to May 2022 using the databases PubMed (https://pubmed.ncbi.nlm.nih.gov/), Web of Science (https://webofknowledge.com), and PsychINFO (https://search.ebscohost.com/). The selected keywords were combined using the Boolean operator AND and OR. The search input was the following: (“personality trait*” OR “impulsivity trait*” OR “Barratt Impulsiveness Scale” OR “Barratt Impulsivity Scale” OR “BIS‐11”) AND (“Parkinson's disease” OR “Parkinson disease” OR “impulse control disorder*”). Additional studies were searched from the references of all identified publications. No language restrictions were applied. Eligibility was determined by a 2‐step procedure performed by 3 of the authors (F.G., C.N., and G.G.). First, the titles and abstracts of all identified articles were screened. In the second step, the full texts of studies, according to predefined eligibility criteria, were independently examined, and agreement was reached after discussion. Our study was conducted following the Preferred Reporting Items for Systematic Reviews and Meta‐Analyses guidelines.^24^

We included controlled studies published in peer‐reviewed journals reporting impulsivity traits assessed by the BIS‐11 in patients with a diagnosis of idiopathic PD and in age‐matched healthy controls or comparing patients with PD with and without ICDs. Included studies had to provide mean and standard deviation (SD) values of the BIS‐11 scores or data to calculate them. Case reports, conference proceedings, and publications available only in abstract form not reporting detailed data were excluded. Studies reporting impulsivity traits assessed by self‐report questionnaires (eg, Eysenck Personality Questionnaire; Eysenck Impulsiveness Questionnaire–I‐7; Dickman Impulsivity Inventory; Lifetime History of Impulsive Behaviors; the Impulsive/Premeditated Aggression Scale; or the more recent Urgency, Premeditation, Perseverance, Sensation Seeking impulsive behavior scale [UPPS]) other than the BIS‐11 were also excluded. We opted for this conservative approach as different self‐report questionnaires developed to assess impulsive traits are based on different theories and models of impulsivity, emphasizing different aspects of this multifaceted psychological construct.

Studies conducted in patients with PD undergoing deep brain stimulation (DBS) were excluded unless the assessments were clearly carried out before the implantation.

### Data Extraction

Data were collected independently by 3 authors (F.G., C.N., and G.G.) using a standardized data extraction form. For each study, the mean and SD of the BIS‐11 total score were extracted or calculated. If available also subscale scores (ie, attentional, motor, and nonplanning) were extracted. BIS‐11 score values were retrieved from text, tables, or estimated by graphs (details are given in Supplementary Material S1). In case of discrepancies, data from tables were chosen. Moreover, authors were contacted to retrieve missing or incomplete data. Other details on data extraction are given in Supplementary Material S1.

In addition, the following data were also extracted for each study: number of participants for each group, mean age, percentage of men, disease duration, Hoehn and Yahr Scale, mean score of the Unified Parkinson Disease Rating Scale (UPDRS) Part III *on* and/or *off* medication, mean levodopa equivalent daily dose (LEDD), and mean dopamine agonist LEDD. Moreover, data on the presence of ICDs in the groups were also extracted. Data were independently extracted and cross‐checked by 3 review authors (F.G., C.N., and G.G.), who also independently assessed the methodological quality of each study.

Data are available from the corresponding author on request.

### Primary Measure

The BIS‐11 is a 30‐item self‐report questionnaire widely used to measure impulsive personality traits.^25^ Each item is measured on a 4‐point Likert scale, with higher values indicating higher impulsivity level. The BIS‐11 includes the following 3 subscales: (1) inability to focus attention or concentrate on the task at hand (attentional impulsivity), (2) tendency to act on the spur of the moment without thinking (motor impulsivity), and (3) lack of planning and forethought (nonplanning impulsivity). Translations of the BIS‐11 are available in several languages.^20^ The internal consistencies (Cronbach's α) reported for the BIS‐11 total score from different translations all fall within an acceptable range (0.71–0.83).^20^ Similarly, the test–retest reliability was acceptable. Therefore, the BIS‐11 is considered a valid tool to assess the construct of impulsiveness in both clinical and nonclinical samples.^20^ A short version of the scale (15 items instead of 30) has been recently validated, and normative data have been provided.^26^ This short version can be used as a quick screening tool to assess impulsivity in a clinical setting. However, to the best of our knowledge, the BIS short version has not been used yet in patients with PD.

### Data Analysis

The meta‐analysis has been conducted using the software RevMan version 5.4.1 (Review Manager, The Cochrane Collaboration, 2020). Mean differences (continuous data) with 95% confidence intervals (CIs) for BIS‐11 total score were separately calculated for studies comparing (1) patients with PD and age‐matched healthy controls and (2) patients with PD with and without ICDs. Included studies reporting BIS‐11 subscale scores (attentional, motor, and nonplanning) were used for a secondary analysis comparing patients with PD with and without ICDs.

Heterogeneity between studies has been assessed by *I* ^2^ and Cochran's Q test. Given the heterogeneity among studies (see the Results), data were analyzed using a random‐effects model.

A weighted least squares linear meta‐regression was performed to explore sources of heterogeneity in the BIS‐11 total score mean difference between patients with PD and healthy controls. The following factors were used as independent variables: percentage of men in the sample, age, disease duration, and LEDD in patients with PD. Because of the rates of missingness across studies, each potential moderator was evaluated in a separate meta‐regression model. To evaluate the influence of LEDD in the mean difference of BIS‐11 total score between patients with PD with and without ICDs, the mean LEDD difference was used as an independent variable in a meta‐regression. The meta‐regression model was weighted by the inverse of variance of each study. The meta‐regression analysis was performed using the software IBM (Armonk, NY) SPSS 20.0; significance was set at *P* < 0.05.

The publication bias has been evaluated by funnel plot inspection. A symmetric funnel plot suggests no publication bias. The presence of asymmetry in the funnel plot was statistically evaluated by Egger's regression asymmetry test using the open‐source software Jeffreys's Amazing Statistics Program ‐ JASP (version 0.16.2; JASP Team 2022, University of Amsterdam, Amsterdam, The Netherlands).

## Results

### Results of the Study Search

**FIG. 1:**
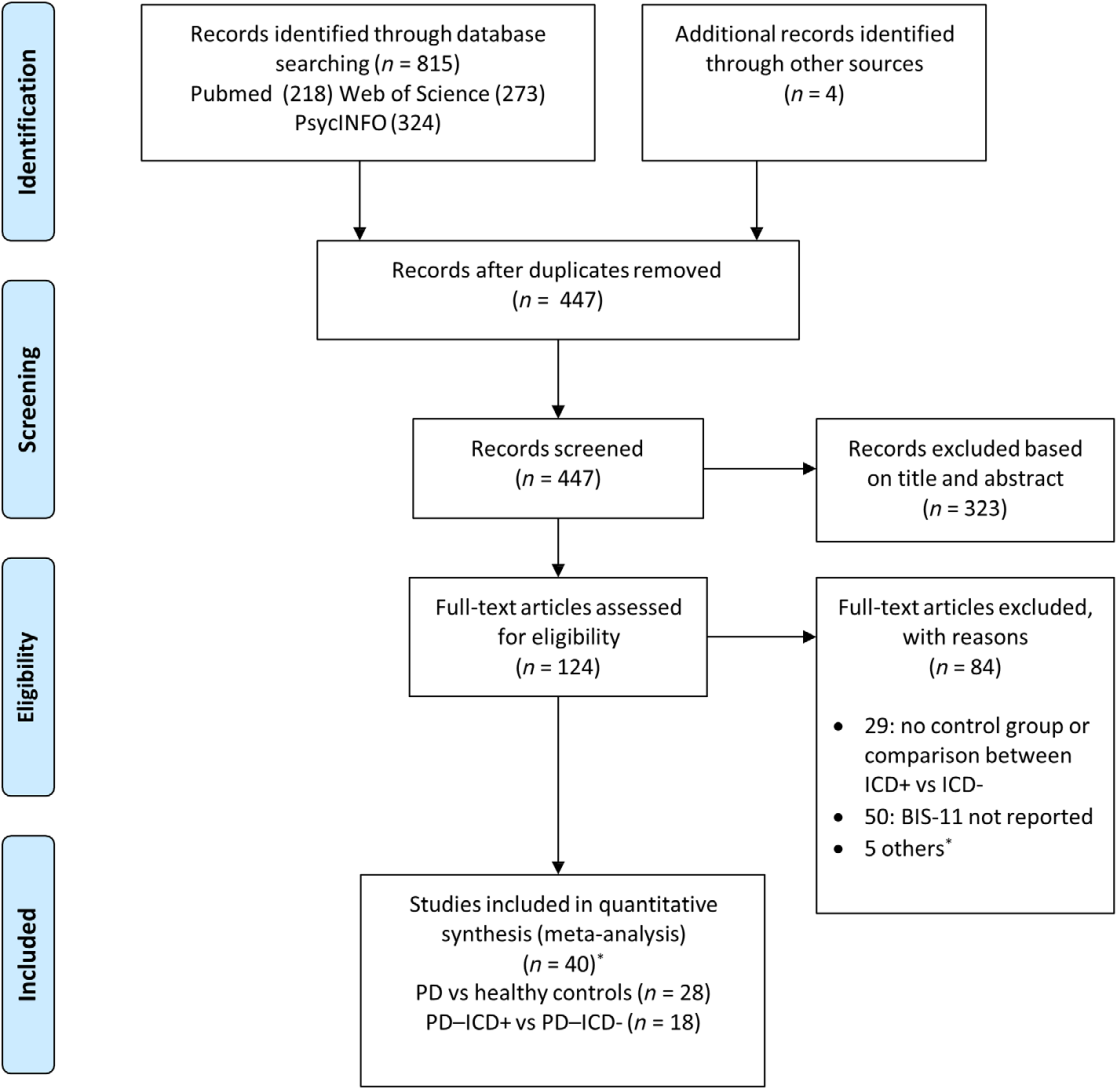
Preferred Reporting Items for Systematic Reviews and Meta‐Analyses flowchart (*see Supplementary Material S1 for details). BIS‐11, Barratt Impulsiveness Scale; ICDs, impulse control disorders; PD, Parkinson's disease.

The flowchart of the article selection is illustrated in Figure 1. Our search yielded 124 potentially eligible studies. After full‐text assessment of these articles, 40 studies (reference list is provided in Supplementary Material S1) from 2007 to 2022 were included in our quantitative analyses (28 studies for the comparison between patients with PD and age‐matched controls and 18 studies for the comparison between patients with PD with and without ICDs). The main characteristics of the studies included in the analysis are reported in Tables 1 and 2. A total of 4 studies were conducted or reported data on newly diagnosed drug‐naïve patients.^19^, ^27^, ^28^, ^29^

**TABLE 1:** Characteristics of studies included in the analysis (patients with PD vs. healthy controls)

Study	Patients with PD									Healthy Controls		
	N	Sex, % Male	Age, y, Mean ± SD or Range	Disease Duration, y	H&Y, Mean or Range	UPDRS III, *On* Medication	UPDRS III, *Off* Medication	LEDD, mg, Mean ± SD or Range	DA‐LEDD, mg, Mean ± SD or Median [Interquartile Range]	N	Age, y, Mean ± SD or Range	Sex, % Male
Chen et al, 2022^15^	50	62	60.4 ± 7.5	NR	NR	NR	27.9 ± 12.5	638 ± 414.5	NR	90	56.2 ± 13.2	51.1
de Chazeron et al, 2021,^32^, ^a^	200	60	67.5 ± 9.9	5.8	NR	16.7 ± 9.8	NR	500 (257–850)	35 [0; 210]	200	67.5 ± 9.9	60
Aumann et al, 2020^14^	68	77.9	65 ± 8.2	5	NR	27.6 ± 12.3	NR	741 ± 411	NR	93	58.0 ± 8.0	53.7
Hlavatá et al, 2020^38^	22	45.5	69.2 ± 5.5	6.95	2.5 ± 0.7	NR	NR	1025.5 ± 567.2	NR	36	58.0 ± 8.1	52.8
Izzo et al, 2020,^33^, ^a^	47	54.9	70.3 ± 7.1	NR	NR	NR	NR	NR	NR	42	69.17 ± 7.5	71.4
Koh et al, 2020^44^	45	40	65 ± 9.2	6.3	NR	23.1 ± 12.8	NR	469 (319–663)	150 (0–285)	21	64.3 ± 10.3	61.9
Pickering et al, 2020^45^	25	68	63.8 ± 5.3	8.1	2 ± 0.6	26.6 ± 12.6	NR	NR	NR	25	68.9 ± 5.6	48
Hammes et al, 2019,^17^, ^a^	62	67.7	68 ± 9.9	4.7	2	NR	25.7 ± 9.9	465 ± 285	142 ± 117	18	67 ± 8.4	44.4
Kubera et al, 2019^46^	22	36.4	64.6 ± 2.2	5.6	1.8 ± 0.6	24.7 ± 10.9	NR	557.2 ± 363.7	NR	18	62.7 ± 2.3	50
Girard et al, 2019^47^	14	100	57 ± 9	6.8	NR	12.6 ± 6.0	28.4 ± 9.1	1068.7 ± 398.8	295.1 ± 161.3	14	54.4 ± 5	100
Picazio et al, 2018^48^	28	50.0	69.0 ± 7.3	9	NR	14.1 ± 3.2	27.4 ± 3.2	591.2 ± 183.1	NR	10	68.3 ± 8.4	50
Aiello et al, 2017^16^	18	66.7	60.2 ± 6.9	9.8	NR	16.5 ± 9	42.7 ± 11.8	1113.9 ± 436.3	NR	18	61.6 ± 8.9	61.1
Duprez et al, 2017^49^	32	56.3	58.7 ± 9.8	9.5	2.7 ± 1.3	11.2 ± 8.9	NR	995.4 ± 316.4	NR	32	55.5 ± 8.9	31.3
Sharp et al, 2016^50^	22	59.1	61.1 ± 6.5	6.8	NR	13.3 ± 6.0	18.6 ± 6.0	715 ± 273	NR	21	62.8 ± 6.8	52.4
Fonoff et al, 2015,^31^, ^a^	28	57.1	59.3 ± 10.3	13.3	2.8 ± 0.6	16.2 ± 7.3	45.5 ± 10.7	1125.6 ± 511.8	NR	28	59.3 ± 11.7	39.3
Grogan et al, 2015^51^	15	NR	71.5 ± 2.4	5.2	NR	19.9 ± 3.2	24.9 ± 3.9	603 ± 71.6	NR	15	71.5 ± 2.6	
Herz et al, 2014^52^	26	57.7	68.2 ± 8.5	6.8	NR	20.6 ± 6.3	32.7 ± 8.7	823.2 ± 371.9	NR	13	68.4 ± 4.9	69.2
Nombela et al, 2014^53^	30	46.7	66.4 ± 10.5	NR	2.2 ± 0.6	23.3 ± 11.1	NR	NR	NR	30	62.4 ± 7.5	46.7
Piray et al, 2014^54^	40	77.5	63.7 ± 3.9	9.4	NR	20.1 ± 5.8	NR	NR	NR	20	66.4 ± 4.7	65
Schomaker et al, 2014^55^	21	71.4	61.8 (51–69)	NR	(2, 3)	21.8	29.1	851.1 ± 581.1	NR	21	60 (49–69)	52.4
Florin et al, 2013^56^	29	100	57.4 ± 9.2	5.3	NR	25.8 ± 7.8	NR	661 ± 528.6	NR	19	56.5 ± 7.2	100
Leroi et al, 2013^57^	55	70.9	62.5 ± 9.2	8.1	2.4 ± 0.7	28.9 ± 13.4	NR	732.2 ± 589.9	156.54 ± 161.11	20	57.9 ± 13.6	55
Rustamov et al, 2013^58^	20	55	58.9 ± 8.3	5.4	2.0 ± 0.9	15.1 ± 6.8	NR	544.4 ± 359.5	NR	20	54.9 ± 4.9	40
van der Vegt et al, 2013^29^	13	61.5	58 ± 10	3	NR	25.6 ± 8.7	NR	Drug‐naïve		12	60 ± 7	41.7
Canesi et al, 2012,^30^, ^a^	36	NR	61 ± 7.5	9.7	2.1 ± 0.4	19.4 ± 8	NR	650 ± 222.6	NR	36	60.2 ± 9.7	NR
Poletti et al, 2012^28^	42	66.7	64.9 ± 7.9	NR	NR	18.2 ± 12.6	NR	Drug‐naïve		30	66.1 ± 7.6	60.0
Cools et al, 2010^59^	15	46.7	64.5 ± 8.5	8.1	NR	13.2 ± 9.8	20.1 ± 11.7	NR	NR	14	66.5 ± 6.2	35.7
Isaias et al, 2008^9^	36	66.7	65 ± 9	8	NR	18.8 ± 6.4	NR	622.0 ± 294.0	NR	80	63 ± 9	50

^a^ Studies including some patients with impulse and compulsive behaviors in the PD group.

Abbreviations: PD, Parkinson's disease; SD, standard deviation; H&Y, Hoehn and Yahr Scale; UPDRS III, Unified Parkinson's Disease Rating Scale–Motor subscale; LEDD, levodopa equivalent daily dose; DA‐LEDD, dopamine agonist levodopa equivalent daily dose; NR, not reported.

**TABLE 2:** Characteristics of studies included in the analysis (ICD positive vs. ICD negative)

Study	PD patients with ICDs								Patients with PD without ICDs							
	N	Sex, % Male	Age, y, Mean ± SD or Range	Disease Duration, y	H&Y, Mean ± SD or Range	UPDRS III, *On* Medication	LEDD, mg, Mean ± SD	DA‐LEDD, mg, Mean ± SD	N	Sex, % Male	Age, y, Mean ± SD or Range	Disease Duration, y	H&Y, Mean ± SD or Range	UPDRS III, *On* Medication	LEDD, mg, Mean ± SD	DA‐LEDD, mg, Mean ± SD
Ricciardi et al, 2021^42^	6	NR	54.6 ± 6.0	8.8	NR	17.0 ± 12.0	852.8 ± 391.3	190.0 ± 119.8	17	NR	60.6 ± 5.4	10.0	NR	19.5 ± 9.4	1112.5 ± 410.8	214.7 ± 166.0
Aumann et al, 2020^14^	43	58.1	60.9 ± 6.9	4.07	NR	25.9 ± 13.1	642.6 ± 397.0	NR	68	77.9	64.9 ± 8.2	5.0	NR	27.6 ± 12.3	740.9 ± 410.6	NR
Hlavatá et al, 2020^38^	15	73.3	59.3 ± 8.9	8.87	2.5 ± 0.6	NR	1289.7 ± 543.9	NR	22	45.5	69.2 ± 5.5	7.0	2.5 ± 0.7	NR	1025.5 ± 567.2	NR
Lee et al, 2019^19^	50	80.0	59.6 ± 9.2	6.55	2.0 ± 0.6	18.5 ± 7.9	545.0 ± 456.3	197.1 ± 118.6	60	43.3	63.9 ± 7.4	7.7	1.9 ± 0.5	17.0 ± 8.5	562.6 ± 264.5	160.4 ± 87.4
Girard et al, 2019^47^	13	100	58.5 ± 8.3	7.5	NR	11.1 ± 5.1	973.1 ± 422.6	282.1 ± 185.1	14	100	57.0 ± 9.0	6.8	NR	12.6 ± 6.0	1068.7 ± 398.8	295.1 ± 161.3
Balconi et al, 2018^60^	15	86.7	65.2 ± 6.3	9.8	2.1 ± 0.6	16.5 ± 8.9	783.1 ± 320.1	NR	17	76.5	60.7 ± 9.1	8.5	1.8 ± 0.8	13.1 ± 7.6	761.9 ± 323.9	NR
Balconi et al, 2018^61^	17	82.4	60.7 ± 6.1	NR	2.0 ± 0.7	17.0 ± 7.8	771.2 ± 320.1	NR	20	85.0	63.9 ± 7.1	NR	1.7 ± 0.5	13.8 ± 7.3	755.7 ± 320.2	NR
Marín‐Lahoz et al, 2018^18^	31	54.8	63.5 ± 9.8	6.48	2 (2–2)	19.9 ± 9.6	702.2 ± 416.9	NR	69	53.6	63.5 ± 9.8	5.5	2 (2–2.5)	23.4 ± 9.9	533.1 ± 451.6	NR
Ruitenberg et al, 2018^62^	21	66.7	60.0 ± 15.0	4.77	(1–3)	25.9 ± 9.9	561.0 ± 322.0	NR	30	63.3	62.0 ± 8.0	3.7	(1–3)	25.3 ± 9.5	486.0 ± 332.0	NR
Pettorruso et al, 2014^63^	34	76.5	62.9 ± 9.6	8.6	NR	19.0 ± 9.7	672.8 ± 373.1	171.4 ± 113.1	120	50.0	67.7 ± 9.4	7.0	NR	20.4 ± 8.4	575.0 ± 420.0	130.0 ± 112.0
Piray et al, 2014^54^	16	87.5	64.4 ± 3.3	9.63	2.47 ± 0.5	19.0 ± 5.3	NR	NR	15	77.5	63.7 ± 3.9	9.4	2.5 ± 0.6	20.1 ± 5.8	NR	NR
Leroi et al, 2013^57^	35	77.1	58.9 ± 9.0	8.04	2.2 ± 0.7	26.8 ± 9.9	994.7 ± 585.4	166.74 ± 175.23	55	70.9	62.5 ± 9.1	8.1	2.4 ± 0.6	28.9 ± 13.4	732.2 ± 589.9	156.54 ± 161.11
Bentivoglio et al, 2013^64^	17	82.4	62 ± 10.1	6.9	2.0 ± 0.8	23.8 ± 11.0	606.1 ± 319.2	172.9 ± 112.2	17	64.7	63.9 ± 9.2	7.3	2.3 ± 0.5	22.5 ± 6.9	616.2 ± 367.8	192.5 ± 88.5
Ray et al, 2012^65^	7		59.7 ± 10.9	10.43	NR	21.0 ± 8.0	888.3 ± 479.9	NR	7	NR	60.6 ± 9.7	8.1	NR	17.1 ± 6.4	644.4 ± 337.7	NR
Antonini et al, 2011^27^	18	83.3	58.3 ± 9.7	1.6	1.4 ± 0.5	16.5 ± 8.1	drug–naïve		85	61.2	60.9 ± 9.1	1.2	1.6 ± 0.5	16.4 ± 8.9	drug–naïve	
Voon et al, 2011^6^, ^13^	282	67.7	60.8 ± 8.4	7.4	NR	19.2 ± 12.4	946.1 ± 609.6	266.3 ± 228.4	282	67.7	61.3 ± 8.4	7.4	NR	19.6 ± 12.4	809.2 ± 609.6	265.2 ± 228.4
Isaias et al, 2008^9^	14	50.0	60.0 ± 9.0	8.5	NR	16.7 ± 6.0	656.0 ± 252.0	NR	36	66.7	65.0 ± 9.0	8.0	NR	18.8 ± 6.4	622.0 ± 294.0	NR
Voon et al, 2007^12^	21	71.4	60.2 ± 8.9	9.2	2.0 ± 0.5	15.2 ± 6.9	874.2 ± 495.6	268.3 ± 194.3	42	50.0	65.7 ± 9.9	6.9	2.2 ± 0.8	22.1 ± 13.9	746.9 ± 322.5	192.1 ± 105.3

Abbreviations: ICD, impulse control disorder; PD, Parkinson's disease; H&Y, Hoehn and Yahr Scale; UPDRS III, Unified Parkinson's Disease Rating Scale–Motor subscale; LEDD, levodopa equivalent daily dose; DA‐LEDD, dopamine agonist‐LEDD; NR, not reported.

### Quantitative Analysis: PD Versus Healthy Controls

**FIG. 2:**
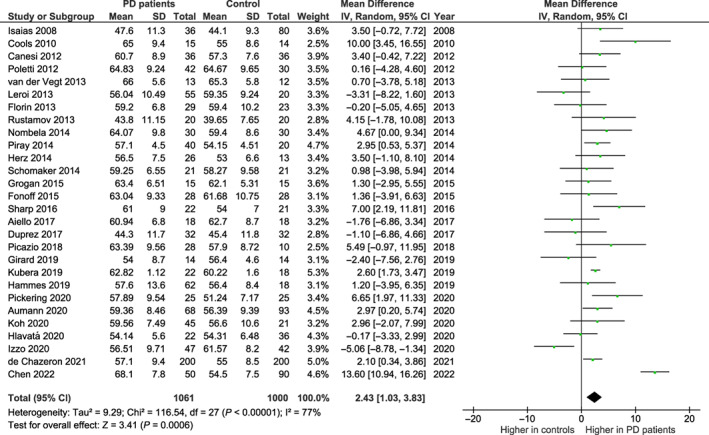
Barratt Impulsiveness Scale total scores in the 28 studies selected for the meta‐analysis comparing patients with Parkinson's disease (PD) to age‐matched healthy controls. CI, confidence interval; df, degree of freedom; SD, standard deviation.

The 28 selected studies included 1061 patients and 1000 healthy age‐matched control subjects. The meta‐analysis revealed a statistically significant mean difference (2.43; 95% CI, 1.03, 3.83), with higher BIS‐11 total scores in patients with PD compared with control subjects (Fig. 2). The heterogeneity was high (*I* ^2^ = 77%, Cochran's Q test *P* < 0.001).

The symmetry of the plots as well as Egger's test suggested no evidence of publication bias for BIS‐11 score mean difference (*z* = −0.371; *P* = 0.711) (Supplementary Material S1).

As a sensitivity analysis, the meta‐analysis was repeated after excluding 5 studies^17^, ^30^, ^31^, ^32^, ^33^ that included in the PD groups some patients with ICDs. Similarly, a statistically significant mean difference (2.85; 95% CI, 1.23, 4.46), with higher BIS‐11 total scores in patients with PD compared with control subjects emerged. The heterogeneity was high (*I* ^2^ = 77%, Cochran's Q test *P* < 0.001).

Meta‐regressions did not reveal statistically significant effects of percentage of men in the sample, age, disease duration, and LEDD as moderators (β = −0.011, F_1,24_ = 0.077, *P* = 0.783; β = −0.287, F_1,26_ = 2.000, *P* = 0.169; β = 0.004, F_1,21_ < 0.001, *P* = 0.985; and β = −0.252, F_1,19_ = 1.290, *P* = 0.270, respectively).

Of 28 studies, 9 reported BIS‐11 subscale scores (Fig. S1). Higher levels of attentional (mean difference: 1.80; 95% CI, 1.54, 2.05) and nonplanning (1.69; 95% CI, 1.31, 2.07) impulsivity emerged for PD patients with ICDs compared with patients without ICDs. No significant difference was observed for the motor impulsivity subscale score (−0.08; 95% CI, −0.42, 0.25).

### Quantitative Analysis: Patients with PD with Versus without ICDs

**FIG. 3:**
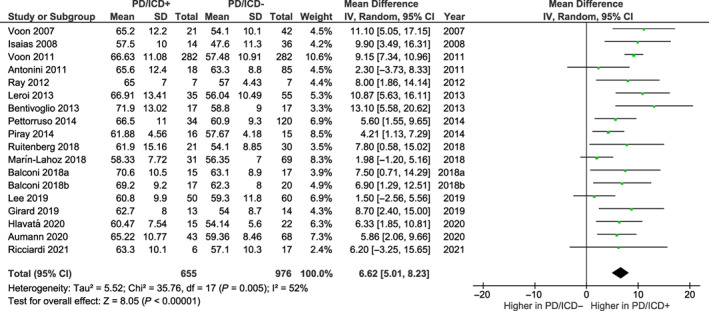
Barratt Impulsiveness Scale total scores in the 18 studies selected for the meta‐analysis comparing patients with Parkinson's disease (PD) with and without impulse control disorders (ICDs). CI, confidence interval; df, degree of freedom; SD, standard deviation.

The 18 selected studies included 655 and 976 patients with and without ICDs, respectively. The meta‐analysis revealed a statistically significant mean difference (6.62; 95% CI, 5.01, 8.23), with higher BIS‐11 total scores in PD patients with ICDs compared with patients without ICDs (Fig. 3). The heterogeneity was moderate (*I* ^2^ = 52%, Cochran's Q test *P* < 0.001).

The symmetry of the plots as well as Egger's test suggested no evidence of publication bias for BIS‐11 score mean difference (*z* = 1.222; *P* = 0.222) (Supplementary Material S1).

When mean LEDD difference between patients with and without ICDs was evaluated as moderator in the meta‐regression analysis, no statistically significant effect was observed (β = 0.163, F_1,15_ = 0.381, *P* = 0.547).

In 8 of 18 studies reporting BIS‐11 subscale scores, higher levels of attentional (mean difference: 1.79; 95% CI, 1.35, 2.22), motor (2.14; 95% CI, 0.96, 3.32), and nonplanning (3.05; 95% CI, 2.36, 3.73) impulsivity emerged for PD patients with ICDs compared with patients without ICDs (Fig. S2).

## Discussion

The 2‐fold aim of the present meta‐analysis was (1) verifying whether impulsivity trait, as assessed by the BIS‐11, is higher in patients with PD with respect to age‐matched healthy individuals and (2) evaluating whether and to what extent the impulsivity traits differ between patients with PD with and without ICDs. The results showed a significantly higher level of impulsivity in patients with PD compared with healthy controls. In addition, BIS‐11 total scores are significantly higher in patients with PD with active ICDs compared with patients without ICDs. Overall, the results of the present meta‐analysis corroborate evidence showing elevated levels of impulsivity in PD, particularly in patients with ICDs.^9^, ^13^, ^14^, ^15^, ^34^

Although the BIS‐11 mean difference between patients with PD and controls was quite small, such a difference remained significant even after excluding studies in which a proportion of patients with PD reported ICDs. It is noteworthy here that in most of the reviewed studies, ICDs are not systematically assessed in the healthy group and, as a consequence, the difference between patients with PD and healthy controls could be underestimated; even more so if we assume that also healthy subjects may have ICDs and related behaviors.^9^

Differences between patients with PD and controls were not significantly influenced by any demographic or clinical factor (ie, percentage of men in the sample, age, disease duration, and LEDD) within the patient groups as revealed by meta‐regression analyses. Particularly noteworthy is that the mean LEDD did not emerge as a significant moderator of the relationship between impulsivity traits and ICDs.

All BIS‐11 domains were higher in patients with PD who were ICD positive compared with ICD negative. In keeping with Aumann et al,^14^ the larger difference between the 2 groups emerged for the nonplanning impulsivity domain. In a recent study,^15^ patients with PD showed elevated scores in all domains of the BIS‐11 compared with healthy controls, whereas patients with cerebellar ataxia exhibited differences in specific domains. Conversely, in the present meta‐analysis, significant differences emerged in the attentional and nonplanning domains, but not in the motor subscale. It must be said, however, that few studies reported the subscale (attentional, motor, and nonplanning) scores. Hence, no robust conclusion can be drawn from the present meta‐analysis on the impulsivity profile characterizing patients with PD.

Among the reviewed articles, 4 studies reported BIS‐11 scores in newly diagnosed drug‐naïve patients, allowing some considerations on the relationship between dopamine replacement therapy and impulsivity traits.^19^, ^27^, ^28^, ^29^ In the study by Antonini et al,^27^ a large sample of drug‐naïve patients with PD were screened for the presence of ICDs and assessed for levels of impulsivity and obsessive‐compulsive symptoms. The proportion of patients who reported at least 1 ICD was 17.5%, a frequency similar to that reported in age‐matched healthy controls.^9^ In patients with PD, the mean BIS‐11 total scores (63.7 ± 9.5; range, 45–91) was below the normative mean values in the age‐matched healthy population.^27^ Patients with PD who were ICD positive showed higher scores in the attentional impulsiveness subscale of the BIS‐11 compared with patients with PD who were ICD negative, with no differences in the total score. Similarly, no differences between patients with de novo PD and healthy controls were reported by Poletti et al^28^ in the BIS‐11 total score (64.8 ± 9.2 vs. 64.7 ± 9.6, respectively). van der Vegt et al^29^ evaluated 13 drug‐naïve patients with PD and 12 healthy age‐matched control subjects who underwent functional magnetic resonance imaging recording during a 2‐choice gambling task. The BIS‐11 total score did not differ between the groups (66.0 ± 5.6 vs. 65.3 ± 5.8 in patients with PD and in healthy controls, respectively). Recently, Lee et al^19^ conducted a multicenter, open‐label trial in which the baseline characteristics of 50 patients with PD with ICD were compared with those of 60 medicated and 40 drug‐naïve PD control groups. The BIS‐11 total score did not differ between the 3 groups of patients. Hence, available data on drug‐naïve patients seem to downsize the role of impulsive personality trait in predicting the risk of developing ICDs. Interestingly, a recent study conducted in patients with de novo PD identified 3 phenotypes based on personality traits and their relationships with motor and neuropsychiatric symptoms.^35^ Impulsivity was observed in the “neuropsychiatric phenotype” characterized by high harm avoidance, low novelty seeking, hypodopaminergic neuropsychiatric symptoms, and higher impulsivity trait. Given the heterogeneity of PD in the early stages, it is conceivable that specific phenotypes may be more associated with the risk of developing ICDs. Moreover, it has been hypothesized that the level of impulsivity may be involved in boosting the severity of ICDs rather than increasing their risk of occurrence.^18^ Reasoning on the results of the present meta‐analysis study, it is evident that a clear definition of the role of impulsivity traits as predisposing factors for the development of ICDs can only be drawn from longitudinal studies. Such studies should aim to assess impulsivity personality profile in patients with de novo PD before starting dopaminergic treatment and to verify longitudinally the incidence of ICDs in individuals with baseline levels of impulsivity exceeding normative values. To the best of our knowledge, no such longitudinal studies have been conducted yet.

A limitation of the present meta‐analysis is that only studies assessing impulsivity traits by the BIS‐11 were selected. This choice may limit the generalizability of the results. However, it should be noted that the literature based on different self‐report tools assessing impulsivity in patients with PD is quite limited for some questionnaires and absent for others.

In a relatively small number of studies, the UPPS was used to assess impulsivity traits in patients with PD instead of or in addition to the BIS‐11. In the study by Bayard et al,^34^ patients with PD without ICDs had greater levels of urgency, lack of premeditation, and lack of perseverance with respect to healthy controls, whereas levels of sensation seeking were higher in patients with ICDs compared with patients without ICDs. Similarly, in some dimensions of the UPPS, higher scores were also reported by Dawson et al^36^ and Olley et al^37^ in patients with ICDs. In contrast, some studies did not observe significant differences between patients with PD and healthy controls in the UPPS scores.^38^, ^39^ Interestingly, Hlavatá et al^38^ reported significant group differences in the BIS‐11 scores but not in the UPPS subscale scores, confirming that different questionnaires evaluate different dimensions of impulsivity.

There is broad consensus that impulsivity is a multidimensional and heterogeneous concept that should not be considered as a unitary construct, instead consisting of a series of independent subtypes reflecting a variety of behaviors and processes.^40^ Accordingly, using voxel‐based morphometry analyses, Marín‐Lahoz et al^41^ showed that different self‐report and behavioral impulsivity measures reflect distinct brain structural correlates. Namely, the impulsivity traits appeared to be associated with lower gray matter volume in the dorsolateral prefrontal cortices. In a recent study conducted in patients who underwent bilateral DBS of the subthalamic nucleus, Ricciardi et al^42^ showed a positive correlation between the oscillatory activity in the α band and the impulsivity traits (BIS‐11 score) in patients with PD, irrespective of the presence and severity of active ICDs. The authors proposed that this spectral feature may represent a neural biomarker associated with impulsive behavior.

In conclusion, the results of the present study support the view that impulsivity as a personality trait may characterize patients with PD even in the absence of ICDs. Moreover, our meta‐analysis corroborates findings of clinical studies reporting higher levels of impulsivity in PD patients with ICDs compared with patients without ICDs. Although the present results broaden the knowledge on the personality profiles of patients with PD,^35^, ^43^ they are currently not exhaustive. Thus, the complex relationship between impulsivity traits and ICDs in PD warrants further investigation.

## Author Roles

(1) Research Project: A. Conception, B. Organization, C. Execution; (2) Statistical Analysis: A. Design, B. Execution, C. Review and Critique; (3) Manuscript Preparation: A. Writing of the First Draft, B. Review and Critique.

F.G.: 1A, 1B, 1C, 2A, 2B, 2C, 3A, 3B

G.G.: 1C, 2A, 2B, 2C, 3A

C.N.: 1C, 2A, 2B, 2C, 3A

P.P.: 1A, 3B

M.P.V.: 1A, 3B

M.C.: 1A, 1B, 1C, 2C, 3A, 3B

## Disclosures

**Ethical Compliance Statement:** The authors confirm that the approval of an institutional review board was not required for this work. The authors confirm that patient consent was not required for this work. We confirm that we have read the Journal's position on issues involved in ethical publication and affirm that this work is consistent with those guidelines.

**Funding Sources and Conflicts of Interest:** No specific funding was received for this work. The authors declare that there are no conflicts of interest relevant to this work.

**Financial Disclosures for the Previous 12 Months:** The authors declare that there are no additional disclosures to report.

## Supporting information


**Supplementary Material S1.** Details on the data extraction, formulas, reference list of studies included in the quantitative analyses, and funnel plots for risk of publication bias.

**Figure S1.** Barratt Impulsiveness Scale–11 subscale (attentional, motor, and nonplanning impulsivity) scores: patients with Parkinson's disease versus healthy controls.

**Figure S2.** Barratt Impulsiveness Scale–11 subscale (attentional, motor, and non‐planning impulsivity) scores: patients with Parkinson's disease with versus without impulse control disorders.
