# Supplementary material for: Impulsivity Traits in Parkinson's Disease: A Systematic Review and Meta‐Analysis
Source: Mov Disord Clin Pract. 2023 Jul 26;10(10):1448–58. doi: 10.1002/mdc3.13839 (PMC10585972; doi:10.1002/mdc3.13839)
Supplement: Supplementary file 1 — Supplementary Material S1. Details on the data extraction, formulas, reference list of studies included in the quantitative analyses, and funnel plots for risk of publication bias. [file MDC3-10-1448-s001.doc]

**Supplementary material 1**

**Details on data extraction**

Antonini et al., 2011: the study was not included in the comparison between PD patients and healthy controls as BIS-11 scores obtained in PD patients were compared to normative mean values in the age-matched healthy population instead to an *ad-hoc* sample.

Aumann et al., 2020 reported BIS-score only as plots, therefore mean and standard deviation data were requested to authors.

Koh et al., 2020: data from PD patients with high (BIS-11 score ≥ 60) and low impulsivity were combined using the formula reported below.

Balconi et al., 2018a and 2018b: only the PD group with actual Pathological gambling was included; the PD group with past pathological gambling was not included in the analysis. Mean disease duration was estimated by subtracting mean age at PD onset from mean age.

Lee et al., 2019: BIS-11 data in PD-ICD and PD non-ICD were estimated by the plot (Figure 3).

Piray et al., 2014: data from PD patients without ICD tested off medication and PD patients without ICD tested on medication were combined using the formula reported below.

Voon 2011: mean disease duration was estimated by subtracting mean age at PD onset from mean age.

Herz et al., 2014: data from PD patients with and without levodopa-induced dyskinesias were combined using the formula reported below.

Picazio et al., 2018 reported BIS-score only as plots, therefore mean and standard deviation data were requested to authors. Data from PD patients with and without levodopa-induced dyskinesias were combined using the formula reported below.

Izzo et al., 2020: data from 47 PD patients who experienced the classical ICDs of gambling, hypersexuality, compulsive eating and compulsive shopping (i.e. excluding PD patients who experienced other ICD-related behaviours such as punding and dopamine dysregulation syndrome) were extracted from Supplementary materials.

**Formula used to calculate standard deviation from standard error**

For studies reporting only standard error, SD values were calculated using the formula:


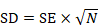


**Formula for combining groups**

|  | **Group 1** | **Group 2** | **Combined groups** |
| --- | --- | --- | --- |
| **Sample size** | N_1_ | N_2_ | 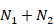 |
| **Mean** | M_1_ | M_2_ | 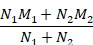 |
| **SD** | SD_1_ | SD_2_ | 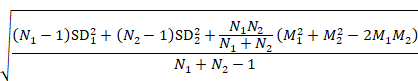 |

Higgins JPT, Li T, Deeks JJ (editors). Chapter 6: Choosing effect measures and computing estimates of effect. In: Higgins JPT, Thomas J, Chandler J, Cumpston M, Li T, Page MJ, Welch VA (editors). Cochrane Handbook for Systematic Reviews of Interventions version 6.3 (updated February 2022). Cochrane, 2022. Available from [www.training.cochrane.org/handbook](http://www.training.cochrane.org/handbook).

**List of studies included in the quantitative analyses**

1. Aiello M, Eleopra R, Foroni F, Rinaldo S, Rumiati RI. Weight gain after STN-DBS: The role of reward sensitivity and impulsivity. Cortex. 2017 Jul;92:150-161. doi: 10.1016/j.cortex.2017.04.005. Epub 2017 Apr 21.
2. Antonini A, Siri C, Santangelo G, Cilia R, Poletti M, Canesi M, Caporali A, Mancini F, Pezzoli G, Ceravolo R, Bonuccelli U, Barone P. Impulsivity and compulsivity in drug-naïve patients with Parkinson's disease. Mov Disord. 2011 Feb 15;26(3):464-8. doi: 10.1002/mds.23501.
3. Aumann MA, Stark AJ, Hughes SB, et al. Self-reported rates of impulsivity in Parkinson's Disease. Ann Clin Transl Neurol 2020; 7 :437-448.
4. Balconi M, Angioletti L, Siri C, Meucci N, Pezzoli G. Gambling behavior in Parkinson's Disease: Impulsivity, reward mechanism and cortical brain oscillations. Psychiatry Res. 2018 Dec;270:974-980. doi: 10.1016/j.psychres.2018.03.041.
5. Balconi M, Siri C, Meucci N, Pezzoli G, Angioletti L. Personality Traits and Cortical Activity Affect Gambling Behavior in Parkinson's Disease. J Parkinsons Dis. 2018;8(2):341-352. doi: 10.3233/JPD-171290.
6. Bentivoglio AR, Baldonero E, Ricciardi L, De Nigris F, Daniele A. Neuropsychological features of patients with Parkinson's disease and impulse control disorders. Neurol Sci. 2013 Jul;34(7):1207-13. doi: 10.1007/s10072-012-1224-5.
7. Canesi M, Rusconi ML, Isaias IU, Pezzoli G. Artistic productivity and creative thinking in Parkinson's disease. Eur J Neurol. 2012 Mar;19(3):468-72. doi: 10.1111/j.1468-1331.2011.03546.x.
8. Chen TX, Lin CR, Aumann MA, et al. Impulsivity Trait Profiles in Patients With Cerebellar Ataxia and Parkinson Disease. Neurology 2022; 99: e176-e186.
9. Cools R, Miyakawa A, Sheridan M, D'Esposito M. Enhanced frontal function in Parkinson's disease. Brain. 2010 Jan;133(Pt 1):225-33. doi: 10.1093/brain/awp301.
10. de Chazeron I, Durif F, Lambert C, Chereau-Boudet I, Fantini ML, Marques A, Derost P, Debilly B, Brousse G, Boirie Y, Llorca PM. A case-control study investigating food addiction in Parkinson patients. Sci Rep. 2021 May 25;11(1):10934. doi: 10.1038/s41598-021-90266-8.
11. Duprez J, Houvenaghel JF, Argaud S, Naudet F, Robert G, Drapier D, Vérin M, Sauleau P. Impulsive oculomotor action selection in Parkinson's disease. Neuropsychologia. 2017 Jan 27;95:250-258. doi: 10.1016/j.neuropsychologia.2016.12.027.
12. Florin E, Müller D, Pfeifer J, Barbe MT, Fink GR, Timmermann L. Subthalamic stimulation modulates self-estimation of patients with Parkinson's disease and induces risk-seeking behaviour. Brain. 2013 Nov;136(Pt 11):3271-81. doi: 10.1093/brain/awt241.
13. Fonoff FC, Fonoff ET, Barbosa ER, Quaranta T, Machado RB, de Andrade DC, Teixeira MJ, Fuentes D. Correlation between impulsivity and executive function in patients with Parkinson disease experiencing depression and anxiety symptoms. J Geriatr Psychiatry Neurol. 2015 Mar;28(1):49-56.
14. Girard R, Obeso I, Thobois S, Park SA, Vidal T, Favre E, Ulla M, Broussolle E, Krack P, Durif F, Dreher JC. Wait and you shall see: sexual delay discounting in hypersexual Parkinson's disease. Brain. 2019 Jan 1;142(1):146-162. doi: 10.1093/brain/awy298.
15. Grogan J, Bogacz R, Tsivos D, Whone A, Coulthard E. Dopamine and Consolidation of Episodic Memory: Timing is Everything. J Cogn Neurosci. 2015 Oct;27(10):2035-50. doi: 10.1162/jocn_a_00840.
16. Hammes J, Theis H, Giehl K, Hoenig MC, Greuel A, Tittgemeyer M, Timmermann L, Fink GR, Drzezga A, Eggers C, van Eimeren T. Dopamine metabolism of the nucleus accumbens and fronto-striatal connectivity modulate impulse control. Brain. 2019 Mar 1;142(3):733-743. doi: 10.1093/brain/awz007.
17. Herz DM, Haagensen BN, Christensen MS, Madsen KH, Rowe JB, Løkkegaard A, Siebner HR. The acute brain response to levodopa heralds dyskinesias in Parkinson disease. Ann Neurol. 2014 Jun;75(6):829-36. doi: 10.1002/ana.24138.
18. Hlavatá P, Linhartová P, Šumec R, Filip P, Světlák M, Baláž M, Kašpárek T, Bareš M. Behavioral and Neuroanatomical Account of Impulsivity in Parkinson's Disease. Front Neurol. 2020 Jan 10;10:1338. doi: 10.3389/fneur.2019.01338.
19. Isaias IU, Siri C, Cilia R, de Gaspari D, Pezzoli G, Antonini A. The relationship between impulsivity and impulse control disorders in Parkinson’s disease. Mov Disord 2008;23:411–415.
20. Izzo VA, Donati MA, Torre E, Ramat S, Primi C. Impulse control disorders in Parkinson's disease versus in healthy controls: A different predictive model. J Neuropsychol. 2020;14:318-332.
21. Koh J, Kaneoke Y, Donishi T, Ishida T, Sakata M, Hiwatani Y, Nakayama Y, Yasui M, Ishiguchi H, Hironishi M, Murata KY, Terada M, Ito H. Increased large-scale inter-network connectivity in relation to impulsivity in Parkinson's disease. Sci Rep. 2020 Jul 10;10(1):11418. doi: 10.1038/s41598-020-68266-x.
22. Kubera KM, Schmitgen MM, Nagel S, Hess K, Herweh C, Hirjak D, Sambataro F, Wolf RC. A search for cortical correlates of trait impulsivity in Parkinson´s disease. Behav Brain Res. 2019 Sep 2;369:111911. doi: 10.1016/j.bbr.2019.111911.
23. Lee JY, Jeon B, Koh SB, Yoon WT, Lee HW, Kwon OD, Kim JW, Kim JM, Ma HI, Kim HT, Baik JS, Cho J; (REIN-PD Investigators). Behavioural and trait changes in parkinsonian patients with impulse control disorder after switching from dopamine agonist to levodopa therapy: results of REIN-PD trial. J Neurol Neurosurg Psychiatry. 2019;90:30-37.
24. Leroi I, Barraclough M, McKie S, Hinvest N, Evans J, Elliott R, McDonald K. Dopaminergic influences on executive function and impulsive behaviour in impulse control disorders in Parkinson's disease. J Neuropsychol. 2013 Sep;7(2):306-25. doi: 10.1111/jnp.12026.
25. Marín-Lahoz J, Pagonabarraga J, Martinez-Horta S, Fernandez de Bobadilla R, Pascual-Sedano B, Pérez-Pérez J, Gironell A, Kulisevsky J. Parkinson's Disease: Impulsivity Does Not Cause Impulse Control Disorders but Boosts Their Severity. Front Psychiatry. 2018 Sep 28;9:465. doi: 10.3389/fpsyt.2018.00465.
26. Nombela C, Rittman T, Robbins TW, Rowe JB. Multiple modes of impulsivity in Parkinson's disease. PLoS One. 2014 Jan 21;9(1):e85747. doi: 10.1371/journal.pone.0085747.
27. Pettorruso M, Martinotti G, Fasano A, Loria G, Di Nicola M, De Risio L, Ricciardi L, Conte G, Janiri L, Bentivoglio AR. Anhedonia in Parkinson's disease patients with and without pathological gambling: a case-control study. Psychiatry Res. 2014 Feb 28;215(2):448-52. doi: 10.1016/j.psychres.2013.12.013.
28. Picazio S, Ponzo V, Caltagirone C, Brusa L, Koch G. Dysfunctional inhibitory control in Parkinson's disease patients with levodopa-induced dyskinesias. J Neurol. 2018 Sep;265(9):2088-2096. doi: 10.1007/s00415-018-8945-1.
29. Pickering JS, Leroi I, McBride J, Poliakoff E. Continuous force measurements reveal no inhibitory control deficits in Parkinson's disease. Exp Brain Res. 2020 May;238(5):1119-1132. doi: 10.1007/s00221-020-05768-0.
30. Piray P, Zeighami Y, Bahrami F, Eissa AM, Hewedi DH, Moustafa AA. Impulse control disorders in Parkinson's disease are associated with dysfunction in stimulus valuation but not action valuation. J Neurosci. 2014 Jun 4;34(23):7814-24. doi: 10.1523/JNEUROSCI.4063-13.2014.
31. Poletti M, Frosini D, Pagni C, Claudio L, Paolo del D, Roberto C, Bonuccelli U. Alexithymia is associated with impulsivity in newly-diagnosed, drug-naïve patients with Parkinson's disease: an affective risk factor for the development of impulse-control disorders? J Neuropsychiatry Clin Neurosci. 2012 Fall;24(4):E36-7. doi: 10.1176/appi.neuropsych.11110326.

1. Ray NJ, Miyasaki JM, Zurowski M, Ko JH, Cho SS, Pellecchia G, Antonelli F, Houle S, Lang AE, Strafella AP. Extrastriatal dopaminergic abnormalities of DA homeostasis in Parkinson's patients with medication-induced pathological gambling: a [11C] FLB-457 and PET study. Neurobiol Dis. 2012 Dec;48(3):519-25. doi: 10.1016/j.nbd.2012.06.021.
2. Ricciardi L, Fischer P, Mostofi A, Tinkhauser G, Torrecillos F, Baig F, Edwards MJ, Pereira EAC, Morgante F, Brown P. Neurophysiological Correlates of Trait Impulsivity in Parkinson's Disease. Mov Disord. 2021;36:2126-2135.
3. Ruitenberg MFL, Wu T, Averbeck BB, Chou KL, Koppelmans V, Seidler RD. Impulsivity in Parkinson's Disease Is Associated With Alterations in Affective and Sensorimotor Striatal Networks. Front Neurol. 2018 Apr 26;9:279. doi: 10.3389/fneur.2018.00279.
4. Rustamov N, Rodriguez-Raecke R, Timm L, Agrawal D, Dressler D, Schrader C, Tacik P, Wegner F, Dengler R, Wittfoth M, Kopp B. Absence of congruency sequence effects reveals neurocognitive inflexibility in Parkinson's disease. Neuropsychologia. 2013 Dec;51(14):2976-87. doi: 10.1016/j.neuropsychologia.2013.10.025.
5. Schomaker J, Berendse HW, Foncke EM, van der Werf YD, van den Heuvel OA, Theeuwes J, Meeter M. Novelty processing and memory formation in Parkinson's disease. Neuropsychologia. 2014 Sep;62:124-36. doi: 10.1016/j.neuropsychologia.2014.07.016.
6. Sharp ME, Foerde K, Daw ND, Shohamy D. Dopamine selectively remediates 'model-based' reward learning: a computational approach. Brain. 2016 Feb;139(Pt 2):355-64. doi: 10.1093/brain/awv347.
7. van der Vegt JP, Hulme OJ, Zittel S, Madsen KH, Weiss MM, Buhmann C, Bloem BR, Münchau A, Siebner HR. Attenuated neural response to gamble outcomes in drug-naive patients with Parkinson's disease. Brain. 2013;136:1192-203.
8. Voon V, Sohr M, Lang AE, Potenza MN, Siderowf AD, Whetteckey J, Weintraub D, Wunderlich GR, Stacy M. Impulse control disorders in Parkinson disease: a multicenter case--control study. Ann Neurol. 2011 Jun;69(6):986-96. doi: 10.1002/ana.22356.
9. Voon V, Thomsen T, Miyasaki JM, de Souza M, Shafro A, Fox SH, Duff-Canning S, Lang AE, Zurowski M. Factors associated with dopaminergic drug-related pathological gambling in Parkinson disease. Arch Neurol. 2007 Feb;64(2):212-6. doi: 10.1001/archneur.64.2.212.

**Figure S1**. Funnel plot: PD vs healthy controls


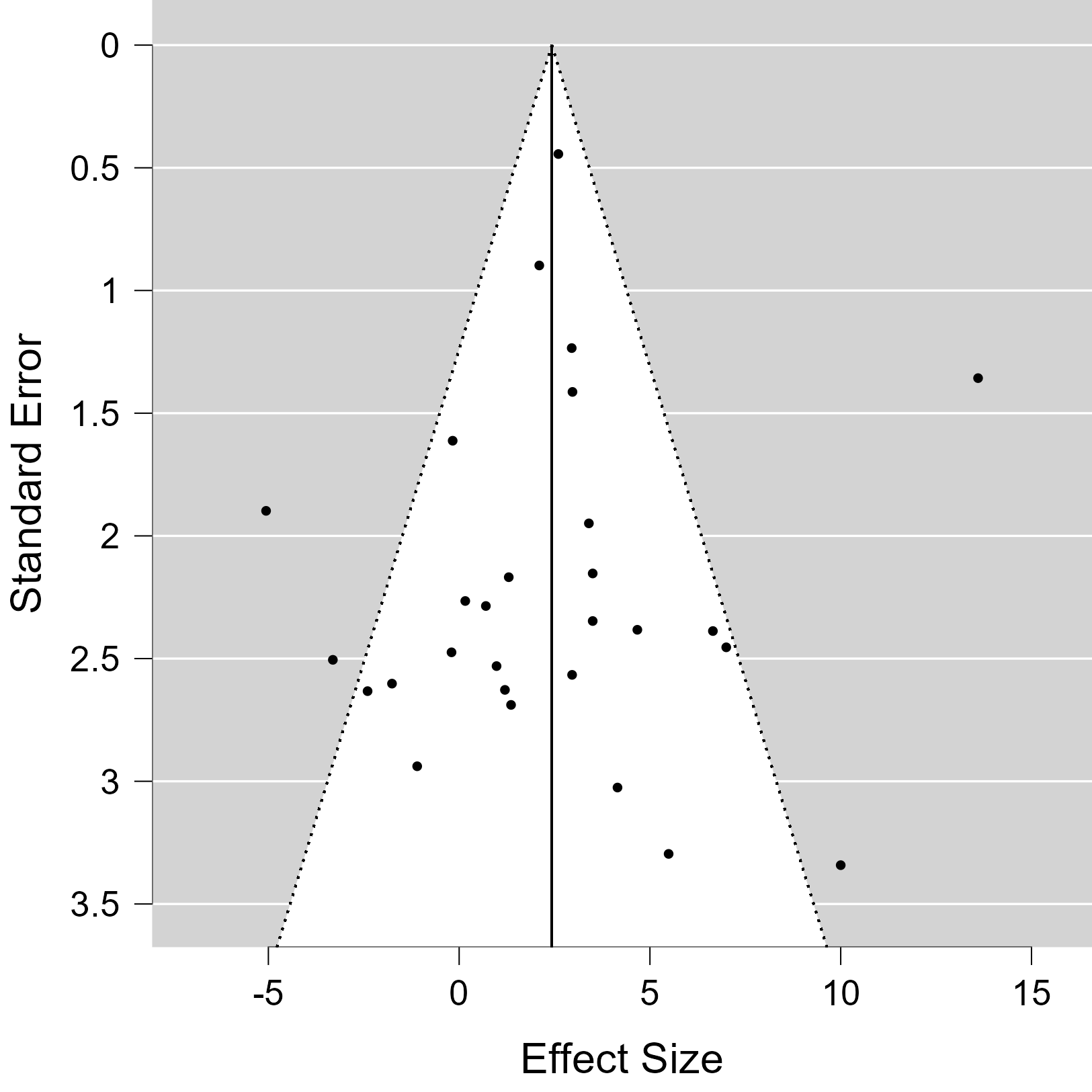


**Figure S2**. Funnel plot: PD patients with vs without ICDs
