# Supplementary material for: Impulsivity Traits in Parkinson's Disease: A Systematic Review and Meta‐Analysis
Source: Mov Disord Clin Pract. 2023 Jul 26;10(10):1448–58. doi: 10.1002/mdc3.13839 (PMC10585972; doi:10.1002/mdc3.13839)

Figure S1. BIS-11 subscale (attentional, motor, and non-planning impulsivity) scores: PD vs healthy controls

Attentional impulsivity subscale

	PD patients			Control				Mean Difference	
Study or Subgroup	Mean	SD	Total	Mean	SD	Total	Weight	IV, Fixed, 95% CI	Year
Fonoff 2015	17.89	3.58	28	16.43	3.56	28	2.0%	1.46 [-0.41, 3.33]	2015
Aiello 2017	16.39	3.7	18	17	3.4	18	1.3%	-0.61 [-2.93, 1.71]	2017
Picazio 2018	16.57	3.35	28	13.9	2.56	10	1.8%	2.67 [0.66, 4.68]	2018
Kubera 2019	16.77	0.59	22	14.39	0.49	18	63.5%	2.38 [2.05, 2.71]	2019
Aumann 2020	13.8	3.24	68	13	3.1	93	7.2%	0.80 [-0.19, 1.79]	2020
Koh 2020	15.27	2.65	45	14	4	21	2.0%	1.27 [-0.61, 3.15]	2020
de Chazeron 2021	16.3	3.6	200	16	3.1	200	16.4%	0.30 [-0.36, 0.96]	2021
Chen 2022	19.16	3.3	50	13.64	3.06	90	5.8%	5.52 [4.41, 6.63]	2022
**Total (95% CI)**			**459**			**478**	**100.0%**	**2.03 [1.76, 2.30]**	

Heterogeneity: Chi² = 80.78, df = 7 (P < 0.00001); I² = 91%
Test for overall effect: Z = 14.92 (P < 0.00001)

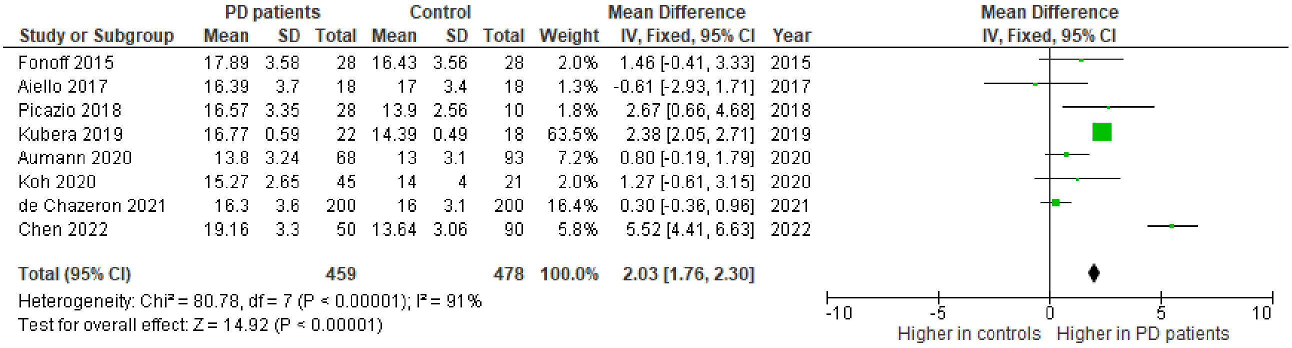

Motor impulsivity subscale

	PD patients			Control				Mean Difference	
Study or Subgroup	Mean	SD	Total	Mean	SD	Total	Weight	IV, Fixed, 95% CI	Year
Fonoff 2015	20.43	3.3	28	19.68	4.22	28	3.0%	0.75 [-1.23, 2.73]	2015
Aiello 2017	20.06	3.4	18	21.7	3.9	18	2.1%	-1.64 [-4.03, 0.75]	2017
Picazio 2018	21.43	3.92	28	20.5	4.67	10	1.1%	0.93 [-2.31, 4.17]	2018
Kubera 2019	21.41	0.68	22	22.11	0.97	18	41.7%	-0.70 [-1.23, -0.17]	2019
Aumann 2020	20.4	2.92	68	21.5	3.51	93	11.9%	-1.10 [-2.10, -0.10]	2020
Koh 2020	20.2	3.37	45	19.8	4.2	21	2.8%	0.40 [-1.65, 2.45]	2020
de Chazeron 2021	17.2	3.8	200	17.1	3.3	200	24.2%	0.10 [-0.60, 0.80]	2021
Chen 2022	22.7	2.8	50	20.07	2.58	90	13.3%	2.63 [1.69, 3.57]	2022
**Total (95% CI)**			**459**			**478**	**100.0%**	**-0.04 [-0.38, 0.30]**	

Heterogeneity: Chi² = 44.20, df = 7 (P < 0.00001); I² = 84%
Test for overall effect: Z = 0.23 (P = 0.82)

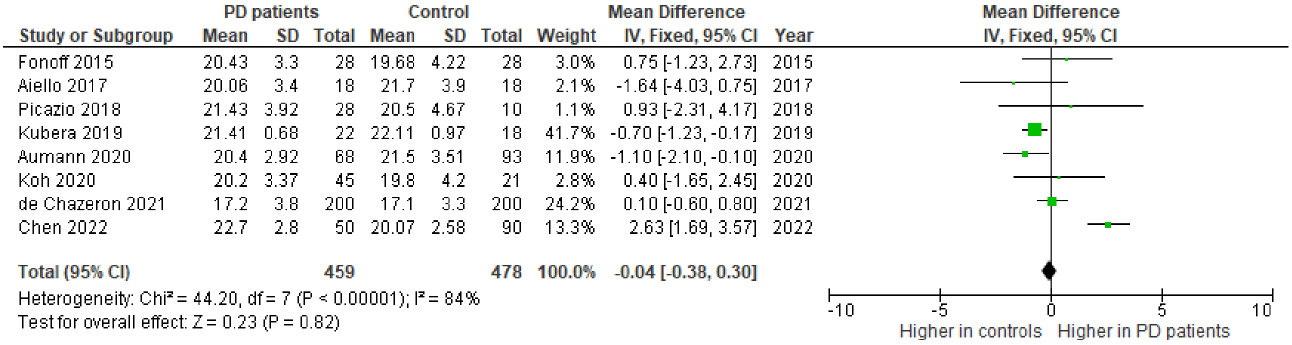

Non-planning impulsivity subscale

	PD patients			Control				Mean Difference	
Study or Subgroup	Mean	SD	Total	Mean	SD	Total	Weight	IV, Fixed, 95% CI	Year
Fonoff 2015	24.71	5.86	28	25.57	5.09	28	1.8%	-0.86 [-3.74, 2.02]	2015
Aiello 2017	24.5	4.3	18	24.06	3.9	18	2.1%	0.44 [-2.24, 3.12]	2017
Picazio 2018	25.39	5.13	28	23.5	4.12	10	1.5%	1.89 [-1.29, 5.07]	2018
Kubera 2019	24.55	0.78	22	22.78	0.81	18	61.2%	1.77 [1.27, 2.27]	2019
Aumann 2020	23.8	6.04	68	21.7	4.56	93	5.2%	2.10 [0.39, 3.81]	2020
Koh 2020	24.13	3.94	45	22.8	5.4	21	2.3%	1.33 [-1.25, 3.91]	2020
de Chazeron 2021	23.5	4.6	200	22	4.2	200	20.2%	1.50 [0.64, 2.36]	2021
Chen 2022	26.2	4.57	50	20.8	4.87	90	5.8%	5.40 [3.78, 7.02]	2022
**Total (95% CI)**			**459**			**478**	**100.0%**	**1.86 [1.47, 2.25]**	

Heterogeneity: Chi² = 23.94, df = 7 (P = 0.001); I² = 71%
Test for overall effect: Z = 9.38 (P < 0.00001)

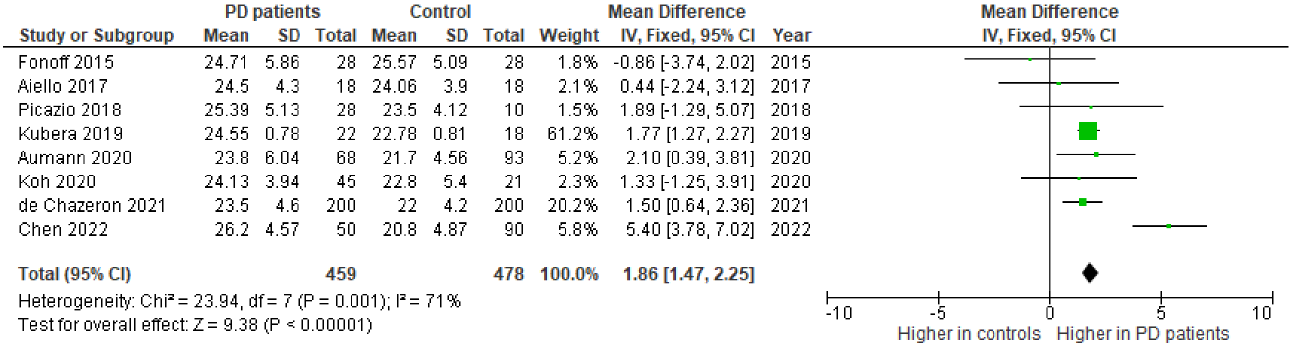

Supplement: Supplementary file 2 — Figure S1. Barratt Impulsiveness Scale–11 subscale (attentional, motor, and nonplanning impulsivity) scores: patients with Parkinson's disease versus healthy controls. [file MDC3-10-1448-s002.pdf]
