# Supplementary material for: Impulsivity Traits in Parkinson's Disease: A Systematic Review and Meta‐Analysis
Source: Mov Disord Clin Pract. 2023 Jul 26;10(10):1448–58. doi: 10.1002/mdc3.13839 (PMC10585972; doi:10.1002/mdc3.13839)

Figure S2. BIS-11 subscale (attentional, motor, and non-planning impulsivity) scores: PD patients with vs without ICDs.

Attentional impulsivity subscale

Study or Subgroup	PD/ICD+ Mean	SD	Total	PD/ICD- Mean	SD	Total	Weight	Mean Difference IV, Fixed, 95% CI	Year
Voon 2007	16.9	3.8	21	15.6	4.1	42	4.5%	1.30 [-0.74, 3.34]	2007
Antonini 2011	18.7	4.9	18	15.2	4.8	85	3.1%	3.50 [1.02, 5.98]	2011
Voon 2011	16.74	3.86	282	14.32	3.86	282	46.5%	2.42 [1.78, 3.06]	2011
Bentivoglio 2013	16.3	5.6	17	12.4	2	17	2.4%	3.90 [1.07, 6.73]	2013
Marín-Lahoz 2018	16.73	1.98	31	16.25	1.98	69	26.8%	0.48 [-0.36, 1.32]	2018
Ruitenberg 2018	16.33	5.25	21	14	3.47	30	2.9%	2.33 [-0.24, 4.90]	2018
Aumann 2020	15	3.28	43	13.8	3.24	68	12.2%	1.20 [-0.05, 2.45]	2020
Ricciardi 2021	17	3.8	6	13.2	3.1	17	1.7%	3.80 [0.42, 7.18]	2021
**Total (95% CI)**			**439**			**610**	**100.0%**	**1.79 [1.35, 2.22]**	

Heterogeneity: Chi² = 19.69, df = 7 (P = 0.006); I² = 64%
Test for overall effect: Z = 8.07 (P < 0.00001)

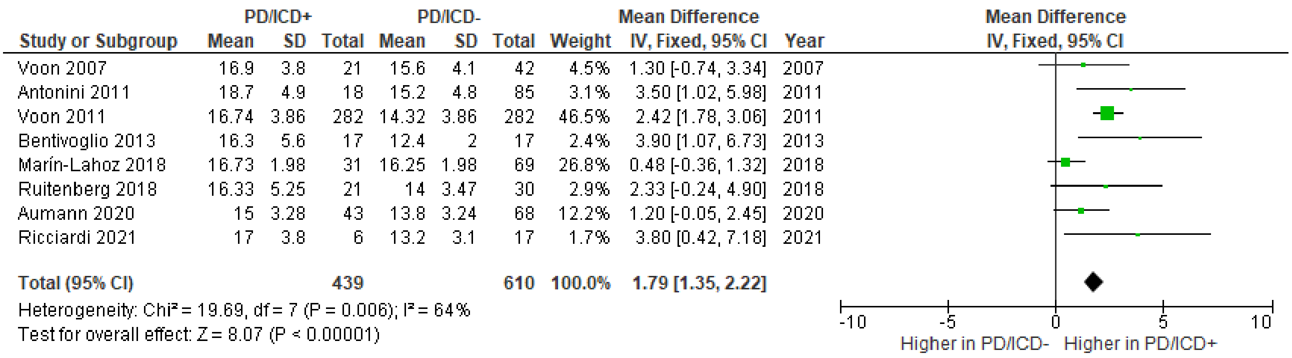

Motor impulsivity subscale

Study or Subgroup	PD/ICD+ Mean	SD	Total	PD/ICD- Mean	SD	Total	Weight	Mean Difference IV, Random, 95% CI	Year
Voon 2007	20.9	5.6	21	17.3	4.4	42	9.5%	3.60 [0.86, 6.34]	2007
Antonini 2011	25.4	5.9	18	24.9	4.4	85	9.0%	0.50 [-2.38, 3.38]	2011
Voon 2011	20.32	4.03	282	17.33	3.86	282	18.3%	2.99 [2.34, 3.64]	2011
Bentivoglio 2013	26	4	17	20.5	4.1	17	9.6%	5.50 [2.78, 8.22]	2013
Marín-Lahoz 2018	19.37	2.95	31	19.31	2.52	69	16.2%	0.06 [-1.14, 1.26]	2018
Ruitenberg 2018	21.9	4.93	21	19.1	2.99	30	10.9%	2.80 [0.44, 5.16]	2018
Aumann 2020	21.8	3.44	43	20.4	2.92	68	16.0%	1.40 [0.16, 2.64]	2020
Ricciardi 2021	22.5	1.9	6	21.2	4.1	17	10.5%	1.30 [-1.17, 3.77]	2021
**Total (95% CI)**			**439**			**610**	**100.0%**	**2.14 [0.96, 3.32]**	

Heterogeneity: Tau² = 1.87; Chi² = 28.23, df = 7 (P = 0.0002); I² = 75%
Test for overall effect: Z = 3.55 (P = 0.0004)

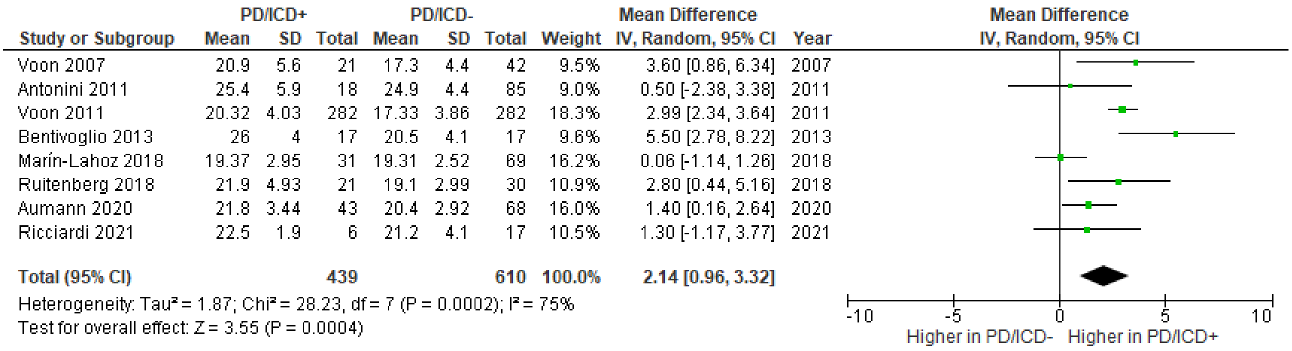

Non-planning impulsivity subscale

Study or Subgroup	PD/ICD+ Mean	SD	Total	PD/ICD- Mean	SD	Total	Weight	Mean Difference IV, Fixed, 95% CI	Year
Voon 2007	27	6	21	21.1	4.6	42	5.5%	5.90 [2.98, 8.82]	2007
Voon 2011	27.25	5.71	282	23.64	5.71	282	53.1%	3.61 [2.67, 4.55]	2011
Antonini 2011	28.1	4.6	18	25.7	4.8	85	8.5%	2.40 [0.04, 4.76]	2011
Bentivoglio 2013	29.6	6.4	17	25.8	5.2	17	3.1%	3.80 [-0.12, 7.72]	2013
Marín-Lahoz 2018	22.23	4.7	31	20.79	4.07	69	12.9%	1.44 [-0.47, 3.35]	2018
Ruitenberg 2018	23.67	6.39	21	21	4.55	30	4.7%	2.67 [-0.51, 5.85]	2018
Aumann 2020	25.25	5.13	43	23.8	6.04	68	10.7%	1.45 [-0.65, 3.55]	2020
Ricciardi 2021	23.8	5.3	6	22.6	6.9	17	1.6%	1.20 [-4.16, 6.56]	2021
**Total (95% CI)**			**439**			**610**	**100.0%**	**3.05 [2.36, 3.73]**	

Heterogeneity: Chi² = 10.91, df = 7 (P = 0.14); I² = 36%
Test for overall effect: Z = 8.70 (P < 0.00001)

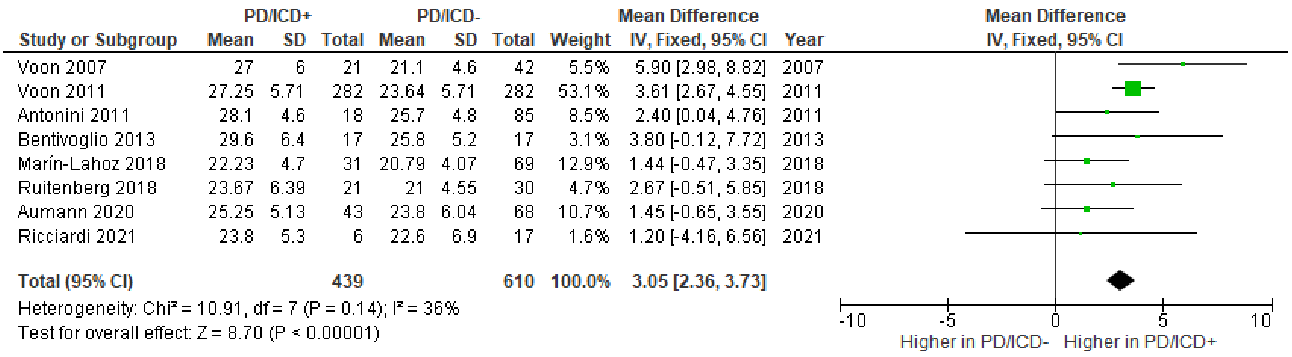

Supplement: Supplementary file 3 — Figure S2. Barratt Impulsiveness Scale–11 subscale (attentional, motor, and non‐planning impulsivity) scores: patients with Parkinson's disease with versus without impulse control disorders. [file MDC3-10-1448-s003.pdf]
